# Next-generation craniomaxillofacial implants for reconstructive surgery: balancing biomechanics, biocompatibility, and bioactivity

**DOI:** 10.1038/s41368-025-00410-7

**Published:** 2026-01-04

**Authors:** Bozhi Hou, Yuehua Li, Raymond Chung Wen Wong

**Affiliations:** National University of Singapore, Faculty of Dentistry, Singapore, Singapore

**Keywords:** Implants, Translational research

## Abstract

Next-generation craniomaxillofacial implants (CMFIs) are redefining personalized bone reconstruction by balancing and optimizing biomechanics, biocompatibility, and bioactivity—the “3Bs”. This review highlights recent progress in implant design, material development, additive manufacturing, and preclinical evaluation. Emerging biomaterials, including bioresorbable polymers, magnesium alloys, and composites with bioactive ceramics, enable patient-specific solutions with improved safety and functionality. Triply periodic minimal surface (TPMS) architectures exemplify how structural design can enhance both mechanical performance and biological integration. Additive manufacturing technologies further allow the fabrication of geometrically complex, customized implants that meet individual anatomical and pathological needs. In parallel, multiscale evaluation techniques—from mechanical testing to in vitro and in vivo models—provide comprehensive insights into implant performance and safety. Looking ahead, the field is poised to benefit from several transformative trends: the development of smart and multifunctional biomaterials; AI-driven design frameworks that leverage patient-specific data and computational modeling; predictive additive manufacturing with real-time quality control; and advanced biological testing platforms for preclinical evaluation. Together, these advances form the foundation of a data-informed, translational pipeline from bench to bedside. Realizing the full potential of next-generation CMFIs will require close interdisciplinary collaboration across materials science, computational engineering, and clinical medicine.

## Introduction

Craniomaxillofacial reconstruction (CMFR) is a critical domain within reconstructive surgery, addressing defects in the skull, face, and jaws caused by trauma, congenital malformations, or tumor resections.^1–3^ The craniomaxillofacial region is uniquely complex, housing vital structures such as the brain, eyes, and airways, while supporting essential functions like chewing, speech, and facial expression. Successful reconstruction demands not only structural restoration but also functional recovery and esthetic harmony to improve patients’ quality of life.^4,5^ Historically, CMFR relied on biological grafts—autografts (e.g., iliac crest bone), allografts, and xenografts—which offer osteoconductive and osteoinductive properties.^6^ However, these approaches face significant limitations: autografts cause donor site morbidity,^7^ allografts risk immune rejection,^8^ and xenografts may transmit diseases.^9^ These challenges, coupled with inconsistent tissue availability, have driven the development of synthetic craniomaxillofacial implants (CMFIs). CMFIs provide customizable, reproducible solutions tailored to individual anatomies, aiming to restore structural integrity, promote osseointegration, and achieve cosmetically acceptable outcomes.^10^

Despite the advancements, current reconstructive CMFIs encounter persistent hurdles. For example, mechanical mismatches with native bone, infection susceptibility, long-term instability of degradation, and poor osseointegration remain prevalent.^11,12^ The craniomaxillofacial region’s exposure to dynamic loads (e.g., masticatory forces) and its proximity to critical anatomical structures amplify these issues, necessitating innovative solutions. Next-generation CMFIs should address these shortcomings, leveraging breakthroughs in biomaterials, design architectures, manufacturing technologies. For instance, growing research interest in biomaterials has been directed toward bioactivity, including osteoinductivity and osseointegration. Novel designs closely emulate the biomechanical characteristics of native bone while ensuring long-term biocompatibility.^13,14^ Additive manufacturing has revolutionized CMFI fabrication, enabling complex porous architectures that enhance osseointegration, mitigate stress shielding, and reduce implant weight.^15,16^

This review synthesizes the state-of-the-art in the early-stage clinical translation of CMFIs in the recent years, including biomaterials selection, design architectures, additive manufacturing technologies, followed by discussion of preclinical evaluation methods and future directions of next-generation CMFIs. Significant attention is devoted to the balance and optimization of biomechanics, biocompatibility, and bioactivity (collectively referred to as the “3Bs” framework) throughout CMFIs development. Key criteria include biomechanical matching,^17^ no toxic or pro-inflammatory effects,^18^ osseointegration potential (chemical cues or coatings to encourage bone bonding).^19^ By examining these facets, we aim to provide a holistic understanding of how next-generation CMFIs are poised to transform CMFR, offering improved outcomes for patients worldwide.

## Early-stage clinical translation and “3Bs” framework

**Fig. 1:**
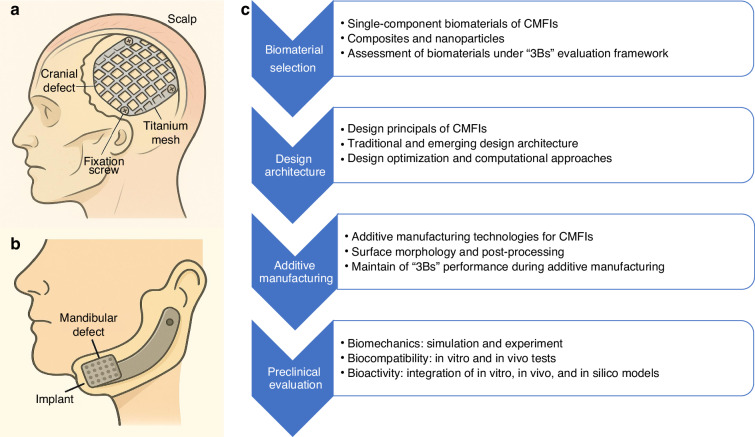
Representative examples of CMFIs and schematic overview of CMFI development. **a** A surgical titanium mesh used for repairing a cranial defect, showing the mesh placement and fixation with screws under the scalp. **b** A patient-specific endoprosthesis for mandibular reconstruction, illustrating implant positioning within the mandibular defect. **c** Schematic overview of the development and evaluation framework for next-generation CMFIs, highlighting the key stages: biomaterials selection, design architecture, additive manufacturing, and preclinical evaluation

The representative examples of CMFIs are shown in Fig. 1a, b, including a titanium mesh for craniofacial reconstruction^20^ and an endoprosthesis for mandibular reconstruction.^21^ However, the clinical translation of patient-specific CMFIs requires a multi-stage process, encompassing material and design development, preclinical validation, manufacturing standardization, regulatory approval, clinical trials, and eventual market deployment and real-world implementation. This review focuses on the early-stage clinical translation, specifically addressing biomaterial selection, design architecture, additive manufacturing, and preclinical evaluation, as highlighted in Fig. 1c. Furthermore, we propose a “3Bs” evaluation framework—encompassing biomechanics, biocompatibility, and bioactivity—and advocate for its integration into the early development pipeline of next-generation CMFIs. This integration ensures next-generation CMFIs undergo comprehensive, multidimensional, and standardized evaluation throughout their development, thereby providing robust clinical support for CMFR surgery. Table 1 presents the “3Bs” evaluation framework proposed in this review, encompassing the specific indices and suggested benchmark applied to the multiscale assessment of CMFIs.

**Table 1:** “3Bs” evaluation framework for CMFIs

Category	Key index	Significance for CMFIs	Suggested benchmark
Biomechanics	Elastic modulus	Determines implant stiffness and affects load transfer to surrounding bone	(0.5–2) × elastic modulus of cortical bone
	Yield strength	Indicates the maximum load the implant can bear before plastic deformation	≥2 × expected max von Mises stress under physiological load
	Fracture toughness	Reflects the implant’s resistance to crack initiation and propagation under the loading	≥10 MPa·m^1/2^ (based on reported properties of practical biomaterials for CMFIs)^202^
	Fatigue Behavior	Describes the implant’s ability to withstand repeated cyclic loading	Endurance ≥ 5 × 10^6^ cycles^203^
Biocompatibility	Inflammatory	Indicates the body’s immune response to the implant (unexpected inflammation lead implant failure)	No persistent chronic inflammation at ≥12 weeks^204^
	Cytotoxicity	Reflects potential harmful effects of implant materials	Cell viability ≥ 70% (100% extract)^205^
	Hemolysis rate	Determine the hemolytic effect on red blood cells	Hemolysis rate (HI) < 2%^206^
	Biodegradation	Refers to the capacity of a material to decompose in vivo over time	Degradation rate < bone regeneration rate
Bioactivity	Osteoconductivity	Supports bone growth along the implant surface	BV/TV ≥ 20% of positive control^207^
	Osteoinductivity	Stimulates new bone formation by inducing stem cell differentiation	ALP activity ≥ 20% of positive control^208^
	Angiogenic potential	Promotes blood vessel formation to nourish regenerating bone	Vessel density > 10 vessels/mm²^209^
	Osseointegration	Ensures stable, direct bonding between bone and implant	Bone-to-implant contact (BIC) > 50%^210^

The suggested benchmarks are indicative and may be adjusted according to implant type and clinical context; measurement methods follow the cited references or ISO standards

**Biomechanics** refers to the study of mechanical principles applied to biological systems. In the context of CMFR, it encompasses the mechanical behavior of facial bones and implants under physiological loads, including stress distribution, deformation, and failure mechanisms. Maintaining or restoring biomechanical integrity is critical to the functional and structural success of CMFIs. The facial skeleton experiences complex loading conditions during mastication, speech, and expression.^22^ Therefore, implants must possess sufficient mechanical strength, stiffness, and fatigue resistance to withstand these forces while mimicking the native bone’s mechanical properties to avoid stress shielding or implant failure. Biomechanical optimization also plays a crucial role in long-term durability and patient-specific implants.^23^

**Biocompatibility** is defined as the ability of a material to perform its desired function without eliciting any undesirable local or systemic effects in the host. It includes cellular response, immune reaction, and toxicity.^24^ In CMFI applications, implants are in intimate contact with bone, soft tissue, and sometimes mucosa, making biocompatibility a fundamental requirement. Poor biocompatibility can lead to inflammation, fibrosis, implant rejection, or infection. Materials must be non-toxic, non-immunogenic, and support tissue healing.^25^ Moreover, high biocompatibility ensures a stable and harmonious interaction between the implant and the host environment, facilitating clinical success and patient safety.^26^

**Bioactivity** refers to the ability of a material to interact with biological tissues at the molecular level to induce specific cellular responses, such as osteogenesis, angiogenesis, or tissue regeneration. Bioactive implants not only serve as structural supports but also actively promote bone regeneration and integration.^27^ In CMFR, this is especially important for large or complex defects where spontaneous healing is limited. Bioactive materials can enhance osseointegration, stimulate osteoblast proliferation and differentiation, and potentially reduce healing time.^28^ Incorporating bioactivity into implant design represents a shift from passive materials toward regenerative solutions, aligning with the principles of tissue engineering and precision medicine.

## Biomaterial selection

This section summarizes both traditional and emerging single-component and composite biomaterials, with parallel consideration given to nanomaterials—such as nanoparticles—that have also been investigated for enhancing biomaterial performance. Representative materials are evaluated with reference to the “3Bs” framework proposed in this study. Biomaterials with adequate literature support for evaluation under the “3Bs” framework are included.

### Single-component biomaterials

Titanium and titanium alloys (e.g., Ti-6Al-4V), as conventional CMFIs, are backed by a comprehensive body of research and long-term clinical evidence.^29–37^ Titanium-based implants exhibit excellent strength, corrosion resistance, osseointegration and MRI-compatibility.^38^ However, its elastic modulus (~110 GPa) far exceeds that of cortical bone (~18–20 GPa), risking stress shielding.^39^ Clinically, titanium meshes and plates are standard for skull reconstruction due to their reliability, but they may require countersinking or contouring to achieve esthetic profiles.^40^ Furthermore, due to its non-biodegradable nature as a permanent implant material, titanium-based implant is not suitable for applications such as pediatric reconstruction and bone tissue scaffolds.^41^ Stainless steel has been historically used in clinical practice; their relatively lower biocompatibility and long-term stability compared to titanium have resulted in reduced usage in recent years. Emerging metal biomaterials such as magnesium alloys are biodegradable implants.^38,42,43^ Magnesium-based CMFIs (e.g., WE43) exhibit excellent biomechanics, as magnesium alloys have an elastic modulus more similar to that of natural bone compared to titanium alloys, thereby reducing the risk of significant stress shielding.^43^ Additionally, magnesium alloys enable gradual in vivo degradation and potentially eliminating the need for secondary surgical removal,^42^ however, controlling rapid corrosion of magnesium alloys remains a challenge. For bioactivity perspective, magnesium alloys releases magnesium ions that may stimulate bone regeneration.^38^

PEEK has also been widely used in clinical practice as a conventional CMFIs polymer.^44–48^ PEEK is a semicrystalline thermoplastic polymer with elastic modulus (~3–4 GPa) much closer to bone than titanium-based implants.^49^ Due to its chemical inertness, PEEK induces negligible inflammatory reactions and cytotoxic effects. Despite its excellent biomechanical properties and biocompatibility, PEEK lacks inherent bioactivity, which limits its ability to promote osseointegration, so surface modification (e.g., hydroxyapatite coating or plasma etching) is often explored to improve osseointegration.^46^ Poly methyl methacrylate (PMMA), as a traditional economical implant material, offers satisfactory mechanical performance^50,51^; nevertheless, its suboptimal biocompatibility and lack of bioactivity.^52^ Polylactide-based homopolymers and copolymers, such as Poly (L-lactic acid) (PLLA), Polylactic-co-glycolic acid (PLGA), and Poly (D, L-lactide) (PLDLLA) have been tried for pediatric CMFIs due to its biodegradability. It can provide temporary support during bone healing, then resorb over months to years. However, they generally have low strength and stiffness,^53^ and degradation products can induce local inflammation. Polycaprolactone (PCL) as an emerging biodegradable polymer exhibits superior biomechanical strength and slow degradation, but its relatively poor bioactivity and long-term persistence may limit its standalone use as CMFIs.^54^

Bioactive ceramics, such as hydroxyapatite (HA) and β-tricalcium phosphate (β-TCP), and bioactive glasses (BAG), such as 45S5 Bioglass and S53P4 Bioglass, are inherently osteoconductive or even osteoinductive.^55^ These materials can support bone cell attachment and stimulate new bone formation, making them highly attractive for regenerative applications.^56^ HA exhibits excellent biocompatibility and osteoconductivity, with a chemical composition closely resembling that of natural bone mineral, making it widely used in coatings and scaffold applications.^57,58^ β-TCP offers excellent osteoconductivity and biodegradability, making it suitable for resorbable bone grafts.^59,60^ 45S5 Bioglass demonstrates excellent bioactivity and strong bonding ability to bone tissue. It degrades gradually in vivo, allowing space for new bone formation without generating toxic byproducts.^61^ However, bioceramics and bioglasses are brittle and typically weaker than metals and alloys, which may not meet the biomechanics requirement as the main material of reconstruction scaffold, especially in load-bearing regions. Therefore, they are more frequently utilized in composites with metals or polymers to capitalize on their superior bioactivity.^62,63^

### Composite biomaterials and nanoparticles

Single-component biomaterials—such as metal alloys, polymers, and ceramics—each exhibit distinct advantages. Nevertheless, none of these materials alone can achieve a comprehensive balance among the “3Bs”. As a result, composite biomaterials have been extensively investigated to achieve comprehensive enhancements in the “3Bs” performance. Alloy-ceramic and polymer-ceramic composites integrate the strengths of the alloys/polymers and ceramics.^64–68^ For example, Ti6Al4V/HA combines the high mechanical strength of Ti6Al4V with the osteoconductivity of HA, promoting stable osseointegration. However, mismatch in elastic modulus and potential delamination of HA coatings may compromise long-term performance. PEEK/HA enhances the bioactivity while maintaining a bone-like elastic modulus suitable for load-bearing applications. exhibiting better cell attachment, proliferation, spreading, and higher alkaline phosphatase (ALP) activity.^64^ Yet, poor interfacial bonding between PEEK and HA remains a challenge for mechanical reliability. PLGA/β-TCP composites offer a balance of biodegradability and bioactivity, making them suitable for non-load-bearing CMFIs, while their limited mechanical strength and potential for acidic degradation byproducts restrict broader application. β-TCP/PLLA/PGA demonstrated significantly enhanced osteogenic cell accumulation compared to pure β-TCP, both at early and late stages of implantation.^67^ But similarly, its poor biomechanical property limits the reliability for load-bearing application.

Nanoparticles have been employed in CMFIs to enhance osseointegration, mechanical performance, and antibacterial properties.^69^ Commonly used nanoparticles include nano-hydroxyapatite (nHA) which mimics the mineral phase of bone and promotes osteogenesis^70^ and titanium dioxide (TiO₂) nanoparticles for surface modification and mechanical reinforcement.^71^ Other promising candidates include zinc oxide and silica-based nanoparticles for their osteoinductive potential, as well as bioactive glass nanoparticles that support both bone formation and angiogenesis.^72^ Carbon-based nanomaterials, such as graphene oxide (GO) and carbon nanotubes (CNTs), offer mechanical benefits and may regulate stem cell behavior. These nanoparticles are commonly integrated into polymer or ceramic matrices, applied as surface coatings, or incorporated into 3D-printed scaffolds to meet the complex demands of CMFR. For instance, PEEK-based bioactive composites reinforced with 0–50 vol% CaO–SiO₂ microspheres, demonstrating enhanced hydroxyapatite-forming ability and improved mechanical properties.^73^ TiO2 incorporated PEEK/HA also enhance the mechanical strength^74^ and bioactivity.^75^ The incorporation of nanoparticles into biocomposite systems has partially overcome the intrinsic limitations of monolithic biomaterials, thereby contributing to the enhanced “3Bs” performance in CMFIs.

### Assessment of biomaterials under “3Bs” evaluation framework

A qualitative evaluation of representative CMFI implant types was conducted using the proposed “3Bs” framework, with the aim of offering preliminary clinical guidance, as detailed in Table 2. The evaluation is based on the criteria provided in Table 1. Taking titanium alloys as an example: its biomechanical performance is rated high (+++) due to its high yield strength, fracture toughness, and fatigue resistance. While a high elastic modulus can contribute to stress shielding, the value remains within an acceptable range when compared to that of stainless steel (~200 GPa), which is substantially higher and thus more prone to causing mechanical mismatch with native bone. Its biocompatibility is also rated high (+++), as the majority of clinical cases have demonstrated that titanium alloys implants are associated with minimal postoperative complications and infections. However, its bioactivity performance is relatively poor (+), since titanium alloys are intrinsically non-osteoinductive.

**Table 2:** Comparison of biomaterials of CMFIs and their “3Bs” performance

Category	Biomaterials	Biomechanics	Biocompatibility	Bioactivity	Applications
Metals/Alloys	Stainless steel	++	+	+	Permanent scaffold, mesh and plate in high load-bearing sites
	Ti alloys	+++	+++	+	Permanent scaffold, mesh and plate in high load-bearing sites
	Mg alloys	++	++	++	Resorbable scaffold and bone substitute
Polymers	PEEK	+++	+++	+	Permanent scaffold in medium load-bearing sites
	PMMA	++	++	+	Emergency surgeries or cost-effective alternative solutions
	PLA copolymers	+	++	+	Resorbable scaffold and bone substitute
	PCL	++	+++	+	Resorbable scaffold and bone substitute
Bioceramics	HA	++	+++	+++	Composite scaffold, coating, filling, and powder forms
	β-TCP	+	+++	+++	Composite scaffold and small-volume bone substitute materials
	Bioglass	+	+++	+++	Bone cement, bone filler, or in combination with titanium mesh
Composite	PEEK/HA	+++	+++	+++	Permanent scaffold in load-bearing sites, suitable for long-term reconstruction scenarios requiring strong osseointegration
	Ti6Al4V/HA	+++	+++	+++	

+ represents the material performs poorly in this property across the board. ++ represents the material is considered acceptable in this property with partial deficiencies. +++ represents the material is widely recognized as a favorable option for this property

## Design architectures

**Fig. 2:**
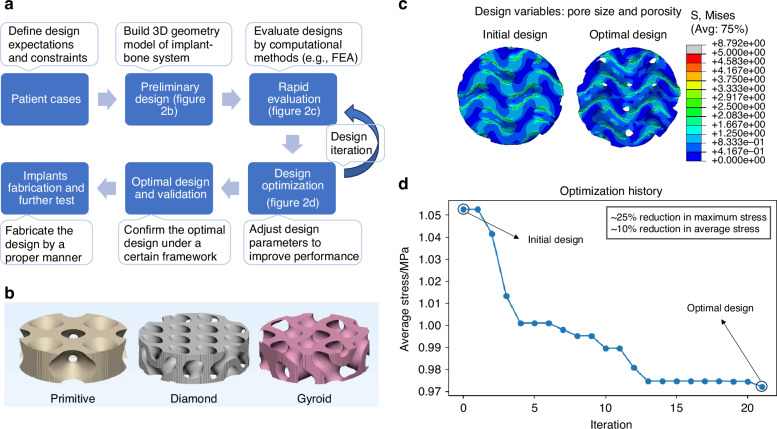
Common design workflow and innovative architectural strategies of CMFIs. **a** Computer-aided design (CAD) workflow for CMFIs. **b** Representative topologies of TPMS-based scaffolds for bone reconstruction. **c** Rapid mechanical evaluation by adjusting porosity and pore size of the scaffolds. **d** Template illustrating the iterative design and optimization process of a TPMS-based scaffold aimed at minimizing stress to prevent potential structural fractures

The success of CMFIs depends not only on material but also on its design architectures, such as morphology, porosity, and fixation pattern. Balancing the “3Bs” properties—biomechanics, biocompatibility, and bioactivity—is a key design principle, encompassing biomechanical matching, anatomical precision, enhancement of osseointegration, and more. Computer-aided design (CAD) accelerates the design iterations of CMFIs. Furthermore, design optimization of the CMFIs can lead to improvements in their “3Bs” performance. This section summarizes the design principles of CMFIs, emerging design architectures, and computational design workflows used to develop the CMFIs that optimize the “3Bs” performance (Fig. 2a).

### Design principles

Given the critical role that biomechanical compatibility plays in the success of CMFIs, the design should match the biomechanical environment of the defect site. For cranial plates and jaw reconstructions, this means sufficient stiffness and strength to withstand physiological loads (e.g. masticatory forces or intracranial pressure) without fracturing, while avoiding stiffness far above native bone.^76^ Design strategies include thickness tuning and lattice structures to adjust stiffness. For example, finite element studies show that increasing plate thickness strongly reduces deflection under load. Conversely, introducing holes or perforations (to promote bone ingrowth) may slightly lower stiffness but often has minimal effect if geometries are optimized.^11^ Computational optimization (e.g. topology optimization) can tailor internal lattices so that stress distribution in an implant mimic that of bone, mitigating stress shielding. Matching fatigue behavior is also important, as jaw implants experience repeated loading; designs must avoid high-strain concentrations.^77^

In addition to mechanical considerations, ensuring precise anatomical conformity is also essential for optimal implant performance. Modern workflow uses patient imaging (CT/MRI) to create 3D models of the defect and contralateral anatomy.^78^ Techniques like mirror-imaging the unaffected side help reconstruct symmetric skull or facial features. CAD tools allow sculpting of the implant geometry for a flush fit against bone.^79^ Good fit reduces dead space (lowering infection risk) and improves mechanical stability by distributing load evenly. Surgical guides can be 3D-printed to ensure accurate placement of screws or osteotomies. Personalization has been shown to reduce operating time and improve outcomes.^80^

Another critical aspect of CMFI design is the promotion of bone ingrowth and integration. To achieve this, the surface characteristics—such as roughness, micro-porosity, and overall bulk porosity—are carefully engineered. For instance, roughened or coated surfaces (e.g., sandblasted Ti, HA coating) increase surface area and cell attachment.^81^ Porous internal architecture provides channels for new bone to invade.^82^ Designs often incorporate scaffolding regions where bone can penetrate through the implant.^83^ The geometry must maintain enough mechanical strength while allowing bone growth. For instance, titanium cranial implants sometimes include perforations to reduce stiffness and enable vascularization, though this must be balanced so as not to weaken the plate excessively.

### Advanced design architecture in CMFIs

Introducing controlled porosity into implants can dramatically improve the 3B performance of CMFIs, as porous scaffolds reduce overall stiffness (bringing it closer to bone) and provide space for vascularized bone ingrowth.^83^ Common porous architectures include open-cell foams and lattices. Random or regular strut lattices (e.g., octet, cubic, or diamond truss) can be 3D printed, allowing tuning of porosity and stiffness. While simple strut lattices are straightforward to design, triply periodic minimal surface (TPMS) geometries offer continuous smooth surfaces, high surface-area-to-volume ratios, and tunable mechanical properties.^84–88^

The design parameters of TPMS structures include unit cell size (pore size), porosity, wall thickness, and topology (e.g. gyroid, diamond, primitive; see Fig. 2b). Pore size is particularly critical: larger pores enhance vascularization and bone ingrowth, whereas smaller pores increase the surface area available for cell attachment.^89^ In practice, intermediate pore sizes (~300–600 μm) are often selected to balance scaffold strength with osteogenic activity.^90–92^ Increasing overall porosity promotes biological infiltration but reduces stiffness, so material selection must be considered together with pore design to achieve optimal osseointegration and mechanical compatibility.^93^ Beyond pore size and porosity, pore shape and interconnectivity also play important roles: rounded or ellipsoidal pores reduce stress concentrations, while the smooth curvature of TPMS structures improves fatigue life.^94^ Because TPMS geometries are governed by mathematical functions, they can be readily generated and computationally optimized to meet balanced “3Bs” performance goals.

To illustrate the “3Bs” framework in implant design, consider three representative cases. First, 3D-printed porous Ti-6Al-4V scaffolds with well-defined circular pores and straightforward interconnected structures (pore sizes ~600 μm) were shown to achieve an optimal balance between biomechanical (mechanical properties analogous to human bone) and biological response (enhanced cell adhesion and proliferation supporting osseointegration).^95^ Second, modified face-centered cubic scaffolds with optimized geometry and porosity exhibited both high mechanical stiffness and efficient cell diffusion, thereby demonstrating that mathematically defined architectures can concurrently fulfill biomechanical and bioactivity requirements.^96^ Third, a topology-optimized titanium layered porous implant for mandibular reconstruction demonstrated that with appropriately tailored porous layout and screw fixation schemes, mechanical stability is maintained (reduced stress shielding) while also providing sufficient mechanical stimulus for bone regrowth according to mechanostat theory.^97^ Together, these implant cases underscore that diverse implant designs can be engineered to balance “3Bs” performance, guiding rational design toward clinical translation.

### Design optimization and computational approaches

Modern CMFIs are designed using advanced computational tools. Patient imaging (CT/MRI) is first segmented to reconstruct the defect 3D geometry. CAD software Design software (often FDA-cleared platforms like Materialise or 3D Systems) enables inverse modeling or manual sculpting of the implant shape. In integrated design platforms, generative optimization algorithms can evolve an initial implant shape into an efficient structure that meets specified mechanical constraints and biological needs. Numerical simulation methods such as finite element analysis (FEA) and computational fluid dynamics (CFD) are routinely used to support the design optimization.^98^ FEA simulates physiological loads to predict stress distribution and implant displacement.^99^ In addition, CFD simulates blood flow inside the porous structure which supports osteoblast function by supplying nutrients and oxygen.^100^ Furthermore, the outcomes of numerical simulations can be leveraged to train predictive models using machine learning techniques, thereby significantly reducing computational costs.^101^ The integration of CAD, computational simulation, machine learning and numerical optimization is the future direction of structural design for CMFIs that achieve both biomechanical efficiency and biological viability.

Designing CMFIs involves navigating a multifaceted landscape of constraints and objectives tailored to patient-specific and site-specific needs, such as defect geometry, anatomical loading, and bone quality.^102^ For optimal biomechanical properties, the designer should find the designs that matches the elastic modulus of native bone (1–20 GPa for cortical bone, 0.1–2 GPa for cancellous bone) to minimize stress shielding.^103^ For enhanced biocompatibility, porosity and pore size are critical variables in the design, as they directly affect permeability and nutrient transport.^100^ Take cranial implants as an example, medium porosity (~50%) ensures structural integrity under intracranial pressure and allows new bone growth.^104^ The design of most CMFIs can be formulated as a constrained, multi-objective, continuous optimization problem—particularly when the design variables are parameterizable, as in the case of TPMS-based structures. To address the inherent trade-offs among competing objectives (the “3Bs” performance), advanced optimization techniques like genetic algorithms and Pareto-front approaches are commonly employed. These facilitate the identification of optimal design solutions that balance multiple performance criteria across diverse clinical scenarios.^98,105,106^

Topology optimization, morphological optimization, and size optimization have been widely applied in the CMFIs design. Topology optimization is crucial for functionally graded TPMS designs because it modifies the internal lattice structure of TPMS-based implants to optimize load distribution, weight, and biological performance.^37,107–110^ For example, it is reported that compared to the uniform implant, the topology-optimized implant showed 20% less stress-shielding and a 45% reduction in the mass.^107^ Morphological optimization adjusts the shape of the pore and hence enhanced the performance. An optimized TPMS design reduces the structural compliance by more than 10.85%, confirming the effectiveness of the proposed optimization framework for lightweight applications.^111^ Size optimization also plays a critical role in the design of CMFIs, particularly in refining parameters such as wall thickness and fixation configurations to enhance mechanical performance and anatomical compatibility. As an illustrative example, the optimization history of a TPMS structure demonstrates that the design converges after 21 iterations by adjusting pore size and porosity (Fig. 2c) to achieve a reduction of approximately 25% in maximum stress and 10% in average stress (Fig. 2d).

## Additive manufacturing

Additive manufacturing (AM), also known as 3D printing, is a technique that builds objects layer by layer from digital models. In the field of CMFIs, AM enables the creation of highly customized implants that precisely match a patient’s anatomy. Surface treatment and post-processing techniques—such as polishing, coating, or heat treatment—are also crucial for improving the biomechanical strength, biocompatibility, and osseointegration of the implants. Together, AM and these post-processing steps ensure that CMFIs are not only anatomically accurate but also safe, durable, and effective in clinical applications. However, the inherent limitations of AM may lead to discrepancies between the actual CMFIs performance and the computational predictions. Therefore, preserving the expected “3Bs” performance throughout the additive manufacturing process is of critical importance.

### AM technologies

Titanium-based implants are frequently fabricated using direct metal laser sintering (DMLS), selective laser melting (SLM) or electron beam melting (EBM). These techniques build parts layer-by-layer by selectively melting or sintering metal powder particles spread across a build platform, enabling the production of complex geometries with controlled internal porosity.^112^ DMLS has been effectively employed in the fabrication of patient-specific implants for the reconstruction of maxillary defects caused by mucormycosis secondary to COVID-19.^113^ SLM offers high resolution and has been successfully applied to manufacture patient-specific implants for 96 cases of primary orbital reconstruction. The results suggested that reconstruction in complex orbital wall fractures achieved with a high degree of accuracy by using SLM-fabricated implants.^114^ In addition, EBM-fabricated cranial implants have demonstrated favorable biomechanics strength and displayed a regular pattern of interconnected channels without any defects and voids, enhancing the esthetic and functional rehabilitation of craniofacial deformities with faster healing and bone ingrowth formation and achieving immediate and efficient reconstruction.^104^ Recent developments have shown the feasibility of AM for Mg alloys based on laser powder bed fusion (L-PBF)^115^ and binder jetting.^116^ However, the risk of combustion during Mg-Zn alloys processing and the inherent corrosion of the Mg-based alloys should also be carefully considered.^117^

Polymers have emerged as promising materials for 3D-printed CMFIs due to their excellent processability and tunable properties. PEEK can be processed using fused filament fabrication (FFF), also known as fused deposition modeling (FDM).^118^ High-temperature FFF systems are capable of melting PEEK and depositing it with precision, enabling the fabrication of implants that closely match CAD geometries. Stereolithography (SLA), selective laser sintering (SLS), and digital light processing (DLP) enable the printing of bioresorbable polymers such as PCL and PLA and have been reported to maintain the biocompatibility of CMFIs.^119^ Polymers can be processed using AM techniques to fabricate complex, patient-specific geometries. For example, 3D-printed PEEK implants have been successfully used in mandibular reconstruction, while PCL scaffolds loaded with growth factors promote bone regeneration in defect sites. Overall, polymer-based 3D printing enables the production of lightweight, precise, and functionally effective implants tailored to individual clinical needs.

The AM of bioceramics scaffolds is primarily accomplished via ceramic SLA/DLP, direct ink writing (DIW, or robocasting), and powder-based methods such as binder jetting. Ceramic SLA/DLP 3D printing enables the fabrication of complex, high-resolution ceramic parts by photopolymerizing a slurry composed of ceramic particles suspended in a photosensitive resin. After printing, the parts undergo sintering to achieve full ceramic density.^120^ In DIW, a viscous ceramic slurry—comprising ceramic particles (e.g., HA, β-TCP, or bioactive glass) suspended in a biopolymer matrix—is extruded through a fine nozzle to form predefined lattice architectures.^121^ The printed structure is then dried and sintered at high temperatures to remove organics and densify the ceramic, yielding porous scaffolds with controlled geometry and interconnected networks favorable for bone ingrowth. In contrast, binder jetting selectively deposits a binder onto a ceramic powder bed to form a “green” part, which is subsequently consolidated via sintering.^120^ In comparison to metals and polymers, the clinical translation of 3D-printed ceramic materials remains relatively limited, primarily due to challenges associated with their brittle nature, high-temperature processing requirements, and complex post-processing procedures.

Current AM-fabricated composite biomaterials for CMFIs are primarily developed using FFF (FDM), DIW, and multi-material jetting technologies. FFF is well-suited for thermoplastic-based composites such as PEEK/HA, enabling the construction of porous architectures with tunable mechanical properties tailored for load-bearing applications.^122^ DIW has been employed to fabricate the Ti/β-TCP scaffolds for bone reconstruction, exhibiting satisfactory biomechanical and biological properties.^123^ Multi-material jetting further expand capabilities by allowing simultaneous deposition of polymers and ceramic, producing multi-materials ceramic parts with high precision, good interfacial bonding and no defects.^68^ However, the application of composite biomaterials in AM-fabricated CMFIs remains limited, with relatively few studies reported to date. This is primarily due to challenges in material compatibility and process control. Differences in thermal and rheological properties among composite constituents’ complicate fabrication, while extrusion- and powder-based AM techniques often suffer from narrow processing windows and poor reproducibility.^124^ Table 3 summarizes representative clinical cases of CMFIs fabricated using various AM technologies over the past five years.

**Table 3:** Case report of CMFIs fabricated by additive manufacturing technologies

CMFIs	Materials	AM Technology	Key conclusions and evaluations
Craniofacial reconstruction	HA	SLA	Optimal bone contact and signs of osteogenesis were observed between the implant and the bone, without donor site morbidity or donor site infection risk.^211^
	Ti6Al4V	SLM	The abnormal intracranial pressure in patients was improvised. There was no evidence of recurrent infection on long-term follow-up_._^212^
	PMMA	FFF/FDM	The functional results and stability of the implants were deemed to be favorable; The rate of surgical revision was zero and without clinically adverse outcomes.^50^
Maxillofacial reconstruction	Ti6Al4V	DMLS	All implants for 20 cases have survived except one which was failed due to wound dehiscence of the implant. And no infection was found on long-term follow-up.^113^
	Ti6Al4V	SLM	The use of SLM implants could prevent possible adverse insertion effects on soft or hard tissues because of sharp edges or displaced mesh.^114^
	BGS-7	FFF/FDM	No donor site morbidity; The implants showed excellent osseointegration and promotes effective bone fusion around the zygomatic bone.^213^
Mandibularreconstruction	Ti6Al4V	SLM	The treatment had excellent postoperative esthetic and functional results without complications.^214^
	PEEK	SLS	All PEEK implants were stable, without displacement, infection, or exposure in 12-24 months follow-up.^215^
	PLDLLA/nHA	FFF/FDM	Follow-up visits confirmed that the implants were non-toxic and biocompatible. For bioactivity, the implants exhibited biomimetic and osteoconductive properties.^216^

### Surface morphology and post-processing

Surface morphology and roughness play a crucial role in the biological performance of additively manufactured implants, influencing early-stage cell adhesion, bacterial colonization, and the generation of wear particles. For example, unprocessed FFF-printed PEEK exhibits a high surface roughness (Ra ~22.28 μm), which can promote osteoblast attachment and growth. Likewise, SLM and EBM typically produce relatively rough surfaces (Ra ~5–20 μm) that support osteoblast adhesion but may also increase the risk of bacterial biofilm formation—particularly in mandibular implants exposed to the oral microbiome.^125^ In contrast, DLP yields significantly smoother surfaces, which are favorable for minimizing neural tissue irritation in cranial applications.^126^ Mechanical surface modifications such as polishing and sandblasting are commonly employed to reduce roughness and improve tissue integration.

A variety of advanced post-processing strategies have been developed to further enhance implant functionality. Electropolishing and plasma polishing can reduce surface roughness by up to 40% and 30% in SLM- and EBM-fabricated implants, respectively, improving both biocompatibility and fatigue resistance.^127^ Ultrasonic cleaning has proven effective in removing up to 95% of residual powder from complex lattice structures such as gyroids, thereby mitigating the risk of chronic inflammation or foreign body response.^128^ Additionally, femtosecond and picosecond laser treatments have emerged as precise methods for creating micro- and nanoscale textures on titanium alloy surfaces, enhancing osteoblast proliferation while reducing bacterial adhesion through modified surface chemistry. Electrochemical deposition of HA coatings—especially those with nanoplates—has also been shown to significantly improve protein adsorption, osteogenic differentiation, and stromal cell proliferation, offering an effective route to biofunctionalized metallic implants.^129^

The integration of these surface modification techniques into the fabrication of AM implants holds significant promise for advancing implant performance. By tailoring surface characteristics at the micro and nanoscale, it is possible to achieve a synergistic enhancement of biological compatibility and mechanical robustness,^130^ paving the way for next-generation CMFIs. Nonetheless, extensive post-processing poses its own challenges. Aggressive surface modification or internal cleaning may inadvertently alter critical pore geometries or reduce structural integrity, particularly in thin-walled or highly porous regions.^131,132^ Therefore, there is a pressing need for standardized, material-specific and geometry-specific post-processing protocols that preserve geometric fidelity.^133,134^

### Challenges in maintaining the “3Bs” in AM

The dimensional accuracy of AM plays a critical role in determining the “3Bs” performance of CMFIs. Deviations from the intended design—resulting from powder adhesion, thermal distortion, or incomplete removal of support structures—can significantly alter pore size, interconnectivity, and overall porosity, thereby impairing osteoconduction, mechanical stability, and osseointegration.^135^ SLM typically achieves dimensional tolerances within ±50 μm, which is generally adequate for mandibular reconstructions requiring mechanical robustness and anatomical conformity. In contrast, electron beam melting (EBM) exhibits slightly lower resolution (±100 μm), necessitating tighter control when used for cranial implants where thin cortical structures and curvature demand higher geometric fidelity.^136^ The results of DIW-based implants showed an overall unsigned dimensional deviation of (30.1 ± 20.2) μm, with a median of 24.4 μm, indicating adequate precision for CMFIs applications.^137^ DLP offers superior accuracy (±20 μm), making it well-suited for intricate craniofacial scaffolds with fine anatomical details.^138^

In addition to dimensional accuracy, each AM techniques exhibit inherent limitations. Metal AM processes typically require extensive post-processing steps—such as stress-relief annealing, precision machining, and surface finishing—to mitigate residual stresses and achieve the desired surface quality.^139^ In polymer-based AM, the layer-by-layer deposition approach often results in anisotropic mechanical properties, primarily due to weaker interlayer bonding compared to the bulk material.^140^ This anisotropy can significantly compromise load-bearing capacity and long-term mechanical durability, particularly in anatomically complex and mechanically demanding applications such as mandibular reconstruction. Bioceramic materials are intrinsically brittle, which constrains the geometric complexity and mechanical robustness of the printed constructs.^141^ Moreover, shrinkage and warping during the drying and sintering stages may lead to the formation of internal defects such as cracks or voids. Achieving adequate mechanical strength in highly porous bioceramics structures remains a significant challenge, especially for load-bearing applications. Additionally, common AM-induced defects—such as voids, incomplete fusion, and irregular microstructures—can serve as stress concentrators, ultimately reducing the structural integrity and reliability of the final implants.^142^

## Preclinical evaluation

**Fig. 3:**
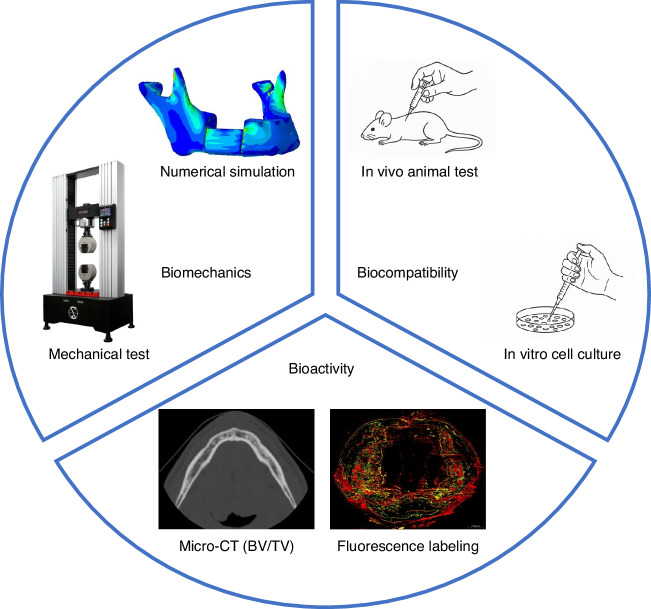
Overview of common preclinical assessment methodologies for CMFIs

To date, there is no end-to-end platform capable of comprehensively evaluating CMFIs across the “3Bs” scales. However, a wide range of mature and emerging methodologies exist for assessing performance within each individual dimension. For instance, biomechanical evaluation can be conducted using finite element analysis and mechanical testing platforms; biocompatibility is commonly assessed through in vitro cell culture and in vivo animal injection models; while bioactivity is often evaluated using techniques such as bone fluorescent labeling and computed tomography imaging (Fig. 3). This section focuses on well-established and widely adopted evaluation methods, laying the groundwork for integrating mainstream assessment techniques into a unified, large-scale development platform in the future.

### Biomechanical assessment

Biomechanical investigations of CMFIs have primarily addressed four aspects: (1) Mechanical strength, including compressive, bending, and torsional properties, which are critical for ensuring the structural integrity and biomechanical stability of implants. (2) Stress shielding, which addresses the need for continuous and physiologically appropriate stress distribution to promote bone regeneration. Studies have demonstrated that the porous titanium mesh induced less stress shielding compared to the dense titanium plate.^104^ (3) Fatigue behavior, which is particularly significant for mandibular and orbital implants subjected to cyclic loading due to masticatory and facial movements. Research highlights that variable amplitude loading can dramatically affect crack growth in the PMMA implants.^143^ (4) Fixation methods, which influence the mechanical stability of the implant-bone interface. For example, comparative analyses suggest that “wing”-type fixation methods produce lower stress concentrations than alternative approaches.^144^ Biomechanical analysis techniques employed in CMFI research can be broadly categorized into experimental mechanics and computational simulations.

For experimental mechanics, tensile and compressive tests on CMFIs are typically conducted using a universal testing machine. Bending tests, such as three-point or four-point bending, evaluate flexural properties. Shear tests assess a material’s resistance to sliding forces, often applied to composites and joints.^51^ Additionally, dynamic mechanical analysis (DMA) and fatigue testing machines are employed to study materials under cyclic loading and time-dependent behavior. Advanced techniques such as Digital Image Correlation (DIC) and micro-Computed Tomography (micro-CT) are increasingly integrated into mechanical experiments.^145^ DIC enables full-field strain measurement by tracking surface deformations in real time, enhancing the understanding of local mechanical responses. Micro-CT provides high-resolution 3D imaging of internal structures, allowing non-destructive evaluation of damage evolution. However, despite their utility, experimental mechanical tests often fall short of replicating the physiological complexity encountered in vivo. Static loading tests do not account for fatigue damage accumulation over time, and fatigue tests with constant amplitude cycles may not adequately represent the variable loading conditions of daily activities.

The Finite Element Method (FEM), a widely recognized numerical approach for mechanical simulations, has long been employed to predict the mechanical behavior of implants under complex loading conditions (e.g., mandible reconstruction), which are often challenging to replicate in experimental setups.^146^ However, FEM typically overlooks the synergistic interactions with biological factors, such as bone-implant integration, tissue ingrowth, and the evolution of mechanical properties during the healing process. Realistic modeling of these interactions necessitates complex multi-scale or growth-adaptive simulations, which, while promising, remain computationally demanding. Thus, recent studies have proposed novel numerical methods to address these challenges. For instance, the integration of musculoskeletal multibody dynamics (MSK-MBD) with FEM has enabled the consideration of muscle and temporomandibular joint (TMJ) influences in the craniomaxillofacial region.^147,148^ Additionally, the combination of machine learning (ML) and FEM has proven effective in reducing computational costs, thus enhancing the efficiency of biomechanical simulations.^149^ However, the accuracy of simulation models still requires comprehensive validations to enable accurate predictions in complex craniomaxillofacial environments.

### Biocompatibility assessment

Ensuring that CMFIs are non-toxic, non-immunogenic, and clinically safe has been the central aim of biocompatibility studies. Standard in vitro evaluation methods include cytotoxicity assays (e.g., MTT, CCK-8), as well as assessments of cell adhesion, proliferation, and inflammatory responses through cytokine profiling.^150^ Recent advanced models such as three-dimensional (3D) co-culture systems, organoid models, and organ-on-a-chip platforms have emerged, providing a more physiologically relevant context by recapitulating cell–cell interactions and tissue architecture. In addition, transcriptomic and proteomic analyses offer molecular-level insights into cellular responses to biomaterials, thereby deepening our understanding of biocompatibility mechanisms. Immune-related in vitro assays, particularly those assessing macrophage polarization (M1/M2), are increasingly employed to evaluate the immunomodulatory properties of candidate materials.^151^ Real-time cell analysis (RTCA) as a dynamic, label-free technique for continuously monitoring cell behavior on biomaterials. This method is particularly advantageous for evaluating biodegradable materials, providing real-time data on cell adhesion and proliferation dynamics.^152^

In vivo biocompatibility assessments involve subcutaneous or intramuscular implantation of materials into animal models to evaluate tissue responses such as inflammation, fibrosis, and immune activation. Histological analysis and systemic immune profiling are commonly employed to assess local and systemic biocompatibility. Critical-sized bone defects are surgically created in the calvarial, mandibular, or orbital regions of animals (typically rats, rabbits, dogs, or sheep) to simulate clinically relevant craniofacial injuries.^153^ Defect sizes vary by species (e.g., 5–8 mm in rats, 8–15 mm in rabbits),^154^ and CMFIs are implanted and monitored over periods ranging from weeks to months. Additionally, multi-omics profiling and single-cell sequencing are being integrated into in vivo studies to dissect the cellular heterogeneity and molecular crosstalk involved in scaffold-mediated bone regeneration.^155^ These approaches provide deeper insight into the mechanisms of immune-bone crosstalk, especially the roles of macrophage polarization, T-cell subsets, and the osteo immunomodulatory environment.

However, several limitations continue to constrain clinical translation. While in vitro assays are indispensable for early screening, they fail to recapitulate the dynamic mechanical loadings, immunologically active, and vascularized environment of living bone.^156,157^ Moreover, most in vivo studies are limited to short-term or medium-term evaluation (typically ≤12 weeks), leaving critical questions about long-term degradation, chronic inflammation, and implant integration unresolved.^158^ The absence of standardized protocols across laboratories—especially regarding defect models, cell types, culture durations, and outcome measures—further complicates comparison and slows clinical translation.^159^

### Bioactivity assessment

Bioactivity evaluations, in contrast, focus on the capacity of implants to actively promote bone regeneration and vascularization. Osteoblast-like cells (e.g., MC3T3-E1 pre-osteoblasts, human mesenchymal stem cells) are typically seeded onto material surfaces or scaffolds to evaluate cellular attachment, viability, and differentiation.^160^ Common assays include viability staining (e.g., MTT, XTT), ALP activity assays, mineralization analysis via Alizarin Red staining,^160^ and the expression of osteogenic markers such as Runx2 and OCN.^161^ Angiogenic potential is evaluated through endothelial cell assays, including tube formation and VEGF expression.^162^ Recently, three-dimensional co-culture models combining osteoblasts and endothelial cells have been developed to better simulate the osteoangiogenic microenvironment and provide more physiologically relevant insights into the bioactivity of scaffolds.^163^ In addition, microfluidic systems and organ-on-a-chip technologies are being explored to replicate the dynamic interplay between bone and vasculature under controlled flow conditions.^164^ These platforms allow for real-time monitoring of cellular behavior and facilitate high-content analysis of biomaterial-induced responses.

In vivo bone regeneration is commonly studied using critical-sized defect models in craniomaxillofacial bones (e.g., calvarial, mandibular, orbital) of rats, rabbits, dogs, or sheep to mimic human clinical conditions.^165^ Genetically modified or immunodeficient animals are increasingly employed to evaluate human cell-based constructs or dissect specific molecular mechanisms. The in vivo bioreactor (IVB) approach, which leverages the body’s intrinsic regenerative capacity through flap prefabrication and axial vascularization, represents a promising direction for personalized bone tissue engineering. Its efficacy has been demonstrated in multiple preclinical models.^166^ Bone healing has been evaluated through histological staining and immunohistochemistry using markers such as osteocalcin (OCN) and CD31.^167,168^ More recently, in silico approaches have been integrated with in vivo animal models to enhance the assessment of bone regeneration. For example, non-invasive imaging modalities—including micro-computed tomography (micro-CT, e.g., bone volume to total volume ratio [BV/TV] analysis), magnetic resonance imaging (MRI), and positron emission tomography-computed tomography (PET-CT)—enable dynamic monitoring of bone formation, scaffold degradation, and host tissue integration.^169^

However, existing methodologies for assessing bioactivity are subject to several inherent challenges. In vitro models, though essential for early-stage screening, often oversimplify the complex osteoimmune and angiogenic environments found in vivo. Traditional 2D cultures fail to replicate the three-dimensional architecture and dynamic cell–matrix interactions critical to bone regeneration.^170^ In vivo animal models, while more physiologically relevant, come with their own constraints. Standard markers such as ALP activity and mineral deposition do not capture the effects of mechanical loading, immune modulation, or nutrient diffusion that influence long-term osseointegration.^171^ Small animals such as rats or rabbits have faster bone regeneration rates and different bone turnover dynamics compared to humans, potentially leading to overestimation of regenerative efficacy.^172–174^

## Next-generation CMFIs

Given the current limitations in biomaterials, design architectures, AM technologies, and preclinical evaluation methods, breakthroughs at multiple levels are urgently needed to develop next-generation CMFIs that achieve balanced and satisfactory performance within the “3Bs” evaluation framework. This review proposes four key directions for future breakthroughs in next-generation CMFIs: (1) Smart and 4D-printing biomaterials; (2) AI-driven design optimization; (3) Predictable models for additive manufacturing; (4) Next-generation platforms for preclinical design and evaluation.

### Smart and 4D-printing biomaterials

**Fig. 4:**
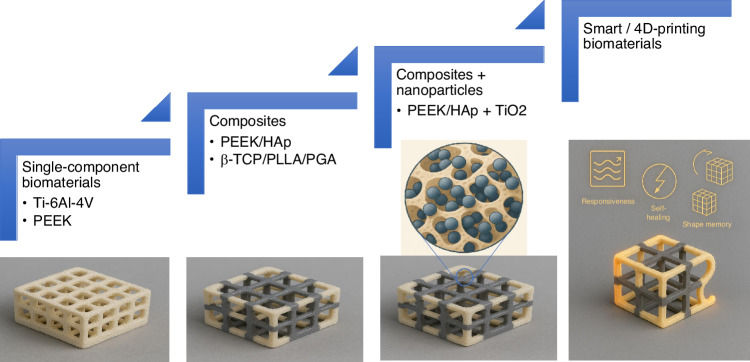
Biomaterials roadmap of CMFIs: from traditional biomaterials to future biomaterials

To date, no single biomaterial has proven capable of fully balancing biomechanics, biocompatibility, and bioactivity. Clinical decision-making requires careful trade-offs. For example, titanium offers high compatibility and stability but severe stress shielding; PEEK offers desired biomechanical performance but is inert. Thus, the development of biomaterials for CMFIs has evolved from the use of single-component materials to composites with nanoparticles. These biomaterials aim to hit the “sweet spot” by combining materials that together meet multiple criteria. For example, titanium meshes was combined with bioactive coatings or bioroot substitutes.^175^ PEEK could be surface-modified or loaded with bioactive particles.^46^ 3D-printed implants with a stiff titanium frame surrounded by a softer, porous polymer-ceramic composite layer. This graded stiffness mimics the transition from cortical bone to marrow or soft tissue. Another concept is an implant with an embedded bioresorbable core and a permanent outer shell. The core provides initial strength and then disappears as bone regenerates.^176^ Fig. 4 shows the development of the biomaterials for CMFIs in recent years.

Recent reviews note that integrating smart materials (e.g., shape-memory polymers (SMPs), magnetically responsive composites) into 3D printed constructs could yield dynamic implants that adapt mechanically or deliver drugs on demand, which is also known as 4D printing biomaterials. 4D printing represents the convergence of additive manufacturing and smart materials, enabling the fabrication of time-dependent, stimulus-responsive implants tailored to dynamic biological environments.^177^ For instance, a compressed Ni-Ti mesh might expand once heated to body temperature, securing itself in a bone bed without extensive screws and enabling minimally invasive implantation.^178^ A folded SMPs-based implants could expand in situ to fit a bone defect.^179^ Future implants may also include bioelectronics (thin-film biosensors) to monitor pressure or strain at the implant–bone interface in real time, alerting clinicians to complications before clinical symptoms appear.^180^ Smart piezoelectric materials scaffolds has also been reported to promote cell proliferation and regeneration of damaged tissues.^181^ Although most of these smart materials remain at the conceptual stage, their integration is anticipated to represent a future trend in the development of CMFIs which comprehensively balance the “3Bs” performance.

### AI-driven design optimization

Design optimization plays a pivotal role in enhancing the “3Bs” performance of CMFIs. However, the extensive design freedom of CMFIs—including variations in materials, geometry, and internal architecture—demands substantial computational resources. Moreover, the design process often demands substantial time investment in CAD modeling and computational simulation, which poses a challenge for timely clinical translation in urgent scenarios. Artificial intelligence (AI) has transformed computational design optimization by enabling efficient exploration of complex, high-dimensional design spaces, by reducing dependence on time-consuming simulations and experimental iterations.^182,183^ The integration of AI to predict and harmonize 3Bs performance presents a transformative strategy for the development of next-generation CMFIs.

Recent efforts have explored the application of AI in the design optimization of CMFIs. Machine learning models, such as convolutional neural networks (CNNs), predict stress distribution, fatigue life, and osseointegration based on TPMS geometry and material inputs of the implants. For examples, a ML model predicted fatigue life of TPMS-based femoral scaffolds with 95% accuracy, minimizing the need for extensive mechanical testing.^101^ Another study uses a machine learning–based model to quickly predict how different factors affect bone growth on 3D-printed biodegradable bone scaffolds. It identifies optimal material and loading conditions to improve bone cell formation, helping to speed up scaffold design.^184^ Additionally, a study demonstrates the potential of AI-optimized Gyroid lattice scaffolds, designed with advanced composites, to enhance mechanical performance and biocompatibility for personalized orthopedic implants.^185^ Although not directly targeting CMFIs, these studies introduce the concept of AI-driven design iteration, enabling real-time, patient-specific design optimization with minimal human intervention and improved implant performance across anatomical sites. Furthermore, leveraging the end-to-end platform presented in Section 6.4, CMFIs design data can be shared among the users, potentially accumulating more data to train the AI model to enable more efficient and accurate AI-driven design optimization.

### Predictable models for additive manufacturing

Although AM technology has revolutionized the fabrication of CMFIs—enabling patient-specific geometries, controlled porosity, and on-demand material deposition—a clear gap remains between the theoretical design models and the actual performance of printed structures.^186^ Variations introduced during printing—such as thermal gradients, microstructural inhomogeneities, surface roughness, and printing resolution limits—can significantly alter the “3Bs” behavior of the final implant.^187^ As a result, many CMFIs based on digital design fail to achieve their intended function in vivo. Consequently, it is a future direction to predict inherent deviations induced by the AM process, and to accordingly adjust the biomaterial selection and design architectures of CMFIs during the pre-design stage, in order to ensure that the manufactured implants achieve the intended performance among the “3Bs”.

To address this, next-generation CMFIs must incorporate process-aware modeling frameworks that explicitly account for the manufacturing process parameters—such as energy input, layer height, scanning speed, and sintering behavior—into performance predictions. For instance, integrated AM–FEM workflows, where printing-induced defects, residual stresses, or local porosity are captured and fed into mechanical or biological simulations, could drastically improve predictive accuracy.^188–190^ In parallel, machine learning models trained on experimental and computational datasets linking AM parameters to final part performance (e.g., compressive strength, degradation rate, bioactivity) offer a promising route to inverse design—where target properties can be used to suggest optimal printing parameters.^191–193^ Additionally, real-time in situ monitoring and imaging techniques (e.g., optical tomography, micro-CT) can provide feedback loops to calibrate and validate predictive models.^194^ Ultimately, bridging the gap between design intent and manufacturing reality will require a convergence of material science, manufacturing process control, and multiscale modeling. This integration will not only improve the reliability and reproducibility of AM implants but also enable true “print-and-predict” capabilities for next-generation CMFIs and patient-specific bone reconstruction.

### Multiscale preclinical design and evaluation platform

**Fig. 5:**
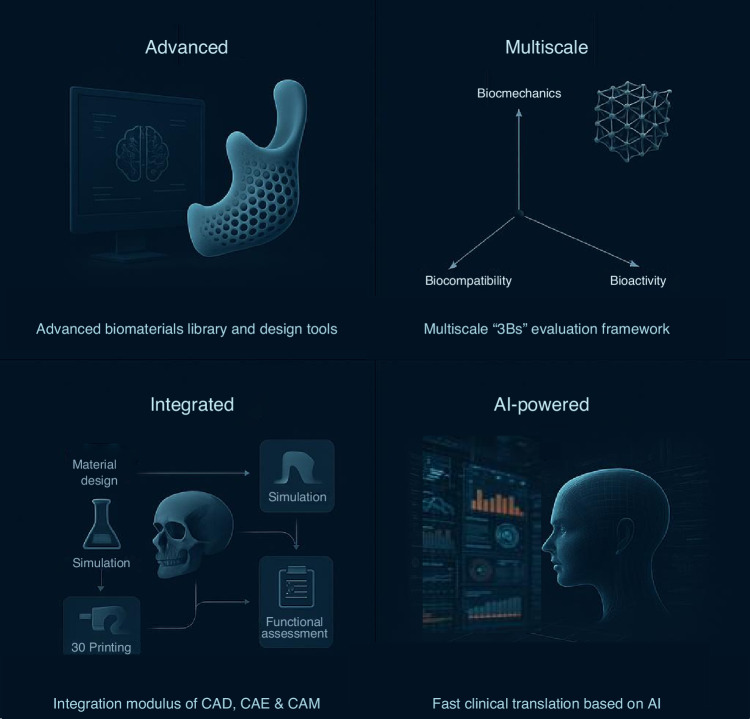
Conceptual diagram of future preclinical design and evaluation platform. CAD computer-aided design, CAE computer-aided engineering, CAM computer-aided manufacturing

Despite significant progress in the development of biomaterials, design architectures and AM technology, current preclinical platforms remain limited in their ability to fully capture the complexity of bone biomechanics (e.g., temporomandibular joint dynamics), patient-specific anatomy, long-term biocompatibility and implant osseointegrations.^195^ Moreover, the current development process of CMFIs typically relies on multiple independent platforms, each utilizing distinct data formats. The interfaces between these steps are often complex and time-consuming. For instance, 3D reconstruction data must be sequentially processed by CAD software, numerical simulation tools, and optimization algorithms. Although certain commercial solutions, such as Materialise, have made substantial efforts to integrate these functions into a unified platform, they still fall short of supporting a fully streamlined design and evaluation workflow. Therefore, next-generation preclinical design and evaluation platform for CMFIs is expected to evolve toward advanced, integrated, multiscale, AI-enhanced, and data-driven frameworks (Fig. 5).

This future platform provides a scalable foundation for the integration of the emerging and high-impact technologies. For example, digital twin will enable real-time, personalized predictions of implant performance, creating a virtual replica of the implant-bone system based on patient-specific imaging, biomechanical data, and biological responses.^196^ A digital twin of a mandibular reconstruction implant can continuously simulate bone ingrowth and stress distribution under masticatory loads, providing early warnings of mechanical failure or poor osseointegration.^197^ Meanwhile, high-throughput experimental platforms, such as automated bioreactor arrays and image-based screening systems, will accelerate in vitro evaluation of materials, topographies, and biological responses.^198^ The convergence of in vivo, in vitro, and in silico approaches will form a closed-loop pipeline, in which computational predictions are validated experimentally and iteratively refined. Furthermore, an emerging bio-chemo-mechanical multiscale model could be used to simultaneously monitor “3Bs” behavior of the implants.^199^ To summarize, this future design and evaluation platform is anticipated to significantly shorten development cycles and improve the safety, efficacy, and individualization of next-generation CMFIs.

### Limitations and challenges for clinical translation of next-generation CMFIs

While Sections “Smart and 4D-Printing Biomaterials”–“Multiscale Preclinical Design and Evaluation Platform” outlined the emerging materials, technologies and platforms that underpin next-generation CMFIs, their translation into clinical practice faces several key challenges. First, regulatory hurdles remain a major bottleneck, particularly for multi-material, 4D-printed, and stimulus-responsive implants, which introduce additional complexity in safety validation, long-term reliability, and risk assessment.^200^ Second, economic and scalability constraints limit widespread adoption. Advanced AM processes, digital twin platforms, and AI-driven design optimization require substantial computational resources, specialized equipment, and expert personnel, making cost-effectiveness and large-scale implementation challenging.^182^ Third, experimental validation and evidence gaps persist: predictive AM models and AI-optimized designs, while highly promising, rely on limited laboratory-scale or small-animal studies, with scarce large-animal or clinical data to confirm safety, efficacy, and generalizability.^201^ Addressing these limitations—including regulatory, economic, and validation-related challenges—will be essential to bridge the gap between experimental innovations and reliable clinical applications of next-generation CMFIs.

## Conclusion

Next-generation CMFIs represent a transformative frontier in personalized craniomaxillofacial bone reconstruction, aiming to optimize the interplay among biomechanics, biocompatibility, and bioactivity—the “3Bs”. This review outlines the current advances in material innovations, design strategies, additive manufacturing, and preclinical evaluation methodologies that collectively drive this evolution. Emerging biomaterials, including bioresorbable polymers, magnesium alloys, bioactive ceramics, and composites with nanoparticles expand the possibilities for patient-specific solutions. Innovative designs, such as TPMS architectures, offer unprecedented control over mechanical properties and biological responses. Additive manufacturing enables the creation of geometrically complex, functional implants tailored to individual anatomical and pathological needs. Concurrently, preclinical evaluation frameworks—spanning multiscale biomechanical testing, biological assays, and in vivo animal models—provide critical insight into the performance of CMFIs.

Looking forward, several frontiers will shape the future of CMFIs development:

- **Smart and 4D-printing biomaterials** will empower implants to dynamically adapt to the evolving biological environment.
- **AI-driven design optimization frameworks** will enable data-informed optimization across structural and biological domains.
- **Predictive models for additive manufacturing** will ensure reproducibility, precision, and customization in CMFIs fabrication.
- **Multiscale preclinical design and evaluation platforms**, integrating advanced multiscale assessing model and AI-enhanced systems, will offer more accurate and reliable evaluation tools, bridging the gap between benchtop and bedside.

Ultimately, a translational pipeline integrating cutting-edge biomaterials science, AI-assisted design, predictive fabrication, and rigorous evaluation will be essential to bring next-generation CMFIs from concept to clinical success. Interdisciplinary collaboration across biomedical engineering, materials science, computational modeling, and clinical practice will be key to realizing the full potential of this promising field.
